# Methods to Evaluate Cell Growth, Viability, and Response to Treatment in a Tissue Engineered Breast Cancer Model

**DOI:** 10.1038/s41598-017-14326-8

**Published:** 2017-10-26

**Authors:** Kayla F. Goliwas, Jillian R. Richter, Hawley C. Pruitt, Lita M. Araysi, Nicholas R. Anderson, Rajeev S. Samant, Susan M. Lobo-Ruppert, Joel L. Berry, Andra R. Frost

**Affiliations:** 1 University of Alabama at Birmingham, Department of Pathology, Birmingham, Alabama, USA; 2 University of Alabama at Birmingham, Department of Surgery, Birmingham, Alabama, USA; 3 University of Alabama at Birmingham, Department of Medicine, Birmingham, Alabama, USA; 4 University of Alabama at Birmingham, Department of Cell, Developmental, and Integrative Biology, Birmingham, Alabama, USA; 5 University of Alabama at Birmingham, Department of Biomedical Engineering, Birmingham, Alabama, USA

## Abstract

The use of *in vitro*, engineered surrogates in the field of cancer research is of interest for studies involving mechanisms of growth and metastasis, and response to therapeutic intervention. While biomimetic surrogates better model human disease, their complex composition and dimensionality make them challenging to evaluate in a real-time manner. This feature has hindered the broad implementation of these models, particularly in drug discovery. Herein, several methods and approaches for the real-time, non-invasive analysis of cell growth and response to treatment in tissue-engineered, three-dimensional models of breast cancer are presented. The tissue-engineered surrogates used to demonstrate these methods consist of breast cancer epithelial cells and fibroblasts within a three dimensional volume of extracellular matrix and are continuously perfused with nutrients via a bioreactor system. Growth of the surrogates over time was measured using optical *in vivo* (IVIS) imaging. Morphologic changes in specific cell populations were evaluated by multi-photon confocal microscopy. Response of the surrogates to treatment with paclitaxel was measured by optical imaging and by analysis of lactate dehydrogenase and caspase-cleaved cytokeratin 18 in the perfused medium. Each method described can be repeatedly performed during culture, allowing for real-time, longitudinal analysis of cell populations within engineered tumor models.

## Introduction

Tissue Engineered (TE) models can be excellent tools for the study of human pathophysiology and disease, which has led to their implementation as *in vitro* models for biomedical and pharmaceutical research^1–5^. TE models of cancer attempt to mimic cancer tissues by including cells and extracellular matrix (ECM) in a realistic three-dimensional (3D) arrangement. The influence of cellular morphology and interactions between adjacent cells and the ECM on cell phenotype and signaling are becoming increasingly well understood with the differences in cell signaling in turn affecting migration, adhesion, gene expression and response to therapeutic intervention^6–14^. Additionally, components of the tumor microenvironment (TME), including stromal cell populations and ECM proteins, have been demonstrated to promote angiogenesis, proliferation, invasion, and metastasis^15–18^. These components can play a functional role in the regulation of cancer progression and resistance to therapeutic intervention^19–21^. Furthermore, therapeutic response is impacted by decreased drug exposure due to the addition of dimensionality that can limit drug diffusion^7,22–24^. These factors may contribute to the observation that many cancer directed therapies that have initially appeared promising in preclinical studies utilizing 2D culture systems have proven to be less effective in 3D systems^22,25–29^. Therefore, therapeutic compounds that target specific molecules or pathways may be better evaluated in 3D TE models, where cellular architecture and the molecular processes described above more closely mimic those found *in vivo*. Together these benefits have promoted the generation of TE model systems for the *in vitro* study of cancer initiation, progression, and response to therapeutic intervention and a variety of TE models have been established to incorporate the complexity associated with human pathologies^1,30–33^.

An important factor for determining the utility of biomimetic, engineered *in vitro* systems for drug screening is their ability to provide real-time feedback and insight into ongoing biological mechanisms and therapeutic response. It is acknowledged that the size, thickness, and complexity of these models can make analysis of cell response to intervention more difficult than analysis of 2D cultures. This is particularly true of analytical methods that allow continued growth after analysis (*i*.*e*., real-time analysis)^34^. Herein, we describe several methods for non-destructive, real-time measurement of cell growth and response to treatment in 3D TE models of cancer. To demonstrate these methods, a perfused, surrogate model of breast cancer was utilized; however, these methods can be adapted for use in other complex, 3D TE model systems.

The perfused breast cancer model utilized differs from most previously generated 3D models of breast cancer in that it approximates the sizes of many human breast cancers (1.0 cm in maximum dimension) at the time of diagnosis, while incorporating relevant components of the TME, including ECM proteins and human cancer-associated fibroblasts (CAF)^35–39^. In a prior report using a similar perfused, 3D surrogate model, our analysis of cell growth over time was limited to terminal endpoint analysis consisting of standard tissue fixation and histologic sectioning^40^. A modified and improved version of the previously described perfused surrogate system was used here to demonstrate several methods of real-time analysis of cell growth and cell death for use in TE models. Some of these methods were originally developed for use in animal models. We also establish the feasibility of using these analytical methods to evaluate real-time response to therapeutic intervention in our breast cancer surrogates.

## Results

### An improved bioreactor design supported the perfusion and growth of *in vitro* 3D breast cancer surrogates

**Figure 1:**
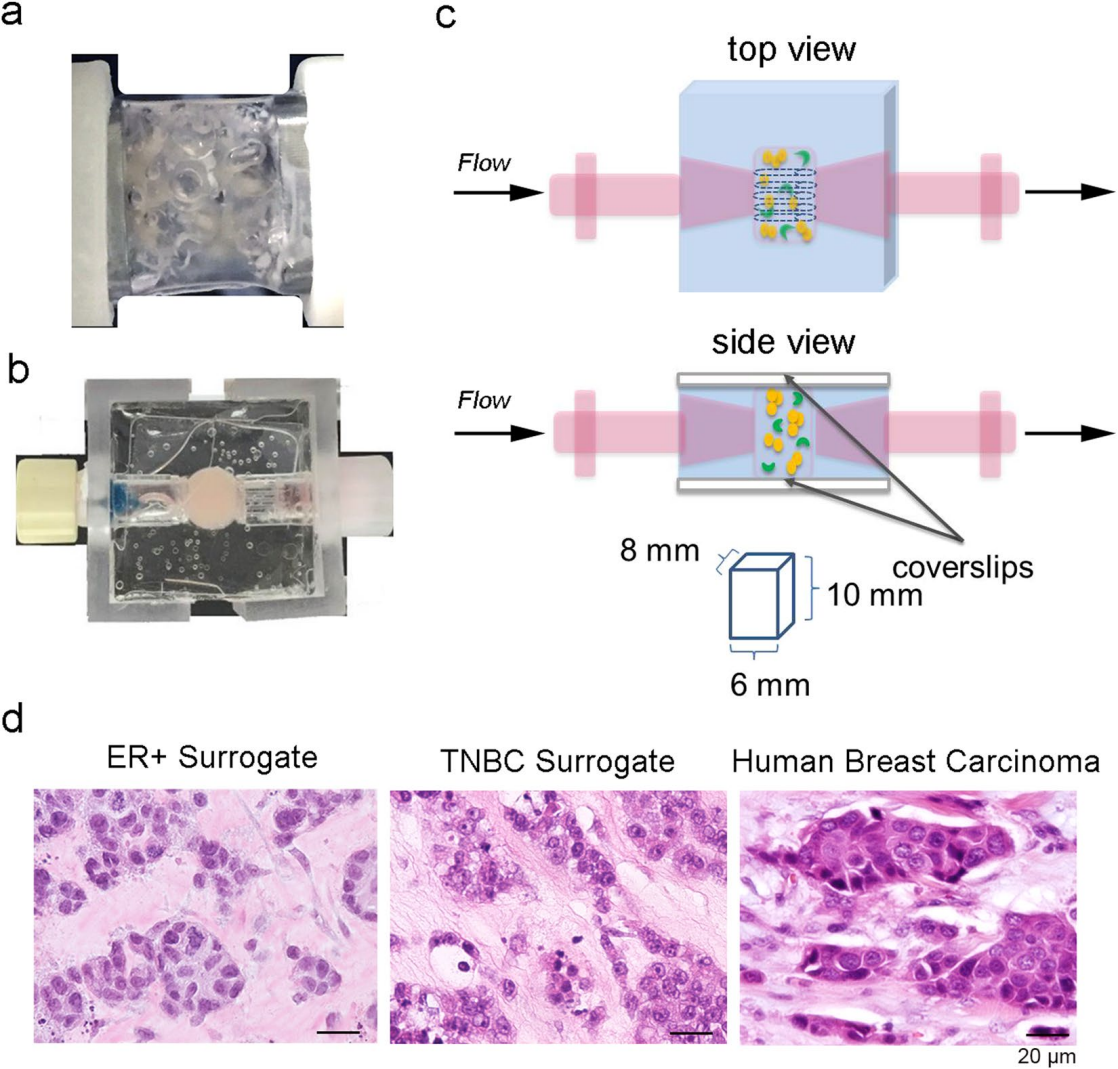
Description of Tissue Engineered Models of Breast Cancer using a Perfusion Bioreactor System. (**a**) Image of the previous bioreactor showing PDMS flow channel containing PDMS foam backbone that hindered non-invasive imaging^41^. (**b**) Top-view photograph of the current bioreactor system showing the optical clarity provided by the coverslips. (**c**) Cartoon representation of the updated breast cancer surrogate containing breast cancer epithelial cells (orange) and cancer associated fibroblasts (green) within a 3D volume of ECM (light pink), all housed within a PDMS bioreactor fabricated to include glass coverslips on the top and bottom surfaces (side view and top view showing microchannels). Surrogate volume (bottom) approximates the sizes of many human breast cancers. (**d**) Photomicrographs of H&E stained histologic sections (200x magnification) from an ER + surrogate (left, culture day 4), a TNBC surrogate (middle, culture day 7) and a human invasive breast cancer (right) demonstrating histologic similarity.

The breast cancer surrogates consist of breast cancer epithelial cells and CAF which are embedded within an ECM, comprised of fibrin, collagen type I, and basement membrane (BM), at a 2:1 ratio of epithelial cells to CAF (as determined in^41^ to be representative of human breast cancer). The engineered surrogates are cultured within a PDMS bioreactor that provides continuous perfusion of medium through 5 microchannels that penetrate the surrogate volume. A prior version of the perfusion bioreactor was previously reported^41,42^ in which a PDMS flow channel contained a PDMS foam. In this version, the cell and ECM surrogate mixture was injected into the PDMS foam and perfused over the span of the experiment (Fig. 1a). This bioreactor provided valuable insight into the maintenance and growth of the engineered surrogates but the PDMS foam that functioned as a structural support hindered long-term growth and real-time imaging. Therefore, the design was modified, as shown in Fig. 1b, to include a wire guide, for uniform generation of through-channels, and glass surfaces for imaging. In contrast to the bioreactor previously reported, the new PDMS bioreactor has a central well (measuring 8 × 6 × 10 mm, Fig. 1c) to contain the surrogates. This perfusion bioreactor system has enabled the generation of models of two breast cancer subtypes, a triple negative subtype model (TNBC) utilizing MDA-MB-231 cells, as previously described^41^, and an estrogen receptor positive (ER+) subtype model utilizing MCF-7 cells. Representative photomicrographs of histologic sections of each of these models demonstrate clusters of the cancer epithelial cells surrounded by the ECM containing scattered, spindled CAF, very similar to the histologic morphology of human breast cancers (Fig. 1d). In addition, we have utilized the surrogate/bioreactor system for *ex vivo* culture of MMTV-neu mouse mammary carcinomas, described below. This TE surrogate system is highly adaptable and can be amended to model other cancers or pathologies. Additionally, other stromal cell components such as immune cell populations and/or endothelial cells could be included to model other aspects of the TME.

### Non-invasive optical imaging measured cancer surrogate growth

**Figure 2:**
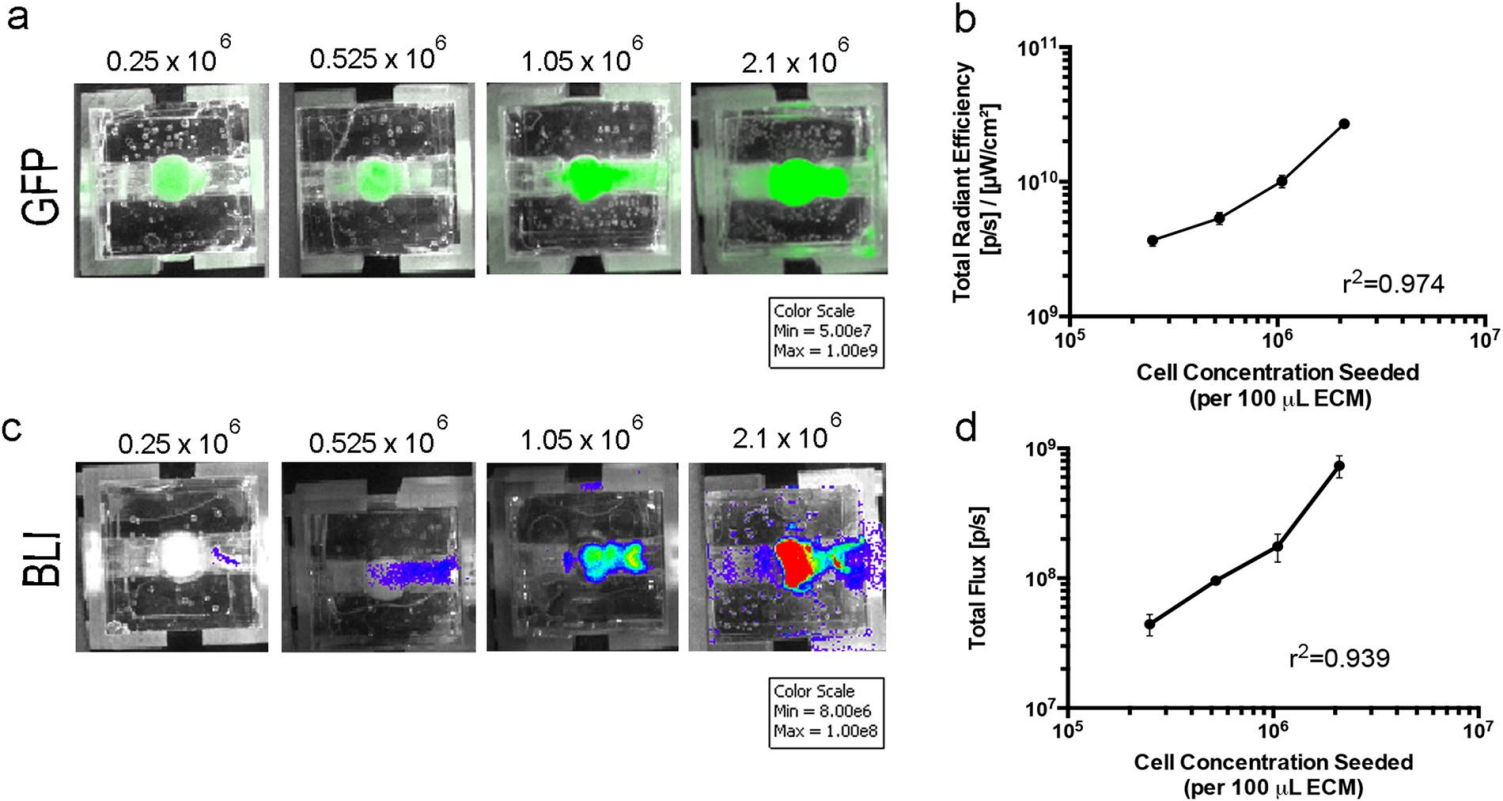
Correlation of Optical Imaging with Cell Concentration within Engineered TNBC Surrogates. (**a**,**c**) Fluorescence (GFP) & bioluminescence imaging (BLI) of increasing cell concentrations (TNBC model) on day 0. (**b**,**d**) Graphical representation of region of interest measurements (ROI) from GFP and BLI completed on the day of surrogate setup (day 0). R^2^ value obtained from correlation analysis of cell concentration seeded and imaging signal shows a strong correlation for both imaging methods (GFP: R^2^ = 0.974 (p = 0.013), BLI: R^2^ = 0.939 (p = 0.031), Pearson correlation coefficient). n = 3–6 replicate surrogates per cell concentration. Data in b & d represent mean ± SE.

The suitability of the non-invasive *in vivo* imaging system, IVIS (Perkin Elmer), to measure global changes in total cell number, as a function of fluorescence or bioluminescence signal, was determined using four increasing concentrations (0.25 × 10^6^, 0.525 × 10^6^, 1.05 × 10^6^, or 2.1 × 10^6^ cells per 100 μL ECM) of MDA-MB-231 breast cancer cells that express both GFP and luciferase. Immediately following ECM polymerization, IVIS imaging was used to measure the intensity of fluorescence (GFP) of each surrogate (1 second exposure, Excitation (Ex): 460/Emission (Em): 520) in regions of interest (ROIs). The same ROI dimensions were used for each surrogate throughout the experiment. The intensity of bioluminescence (BLI) in ROIs was also measured in a similar manner after injection of d-luciferin (1 mL, 5 μg/mL) into the surrogate volume from an upstream port and an 8 minute incubation. Both GFP (Fig. 2a) and BLI (Fig. 2c) signals increased as the cell concentration increased. These increases in signal intensity correlated strongly with the cell seeding concentrations (r^2^ = 0.97 (GFP), r^2^ = 0.94 (BLI), Pearson’s correlation testing, n = 3–6), as shown in Fig. 2b and d, respectively. Similar results were found in the ER+ model (see Supplementary Fig. S1).

**Figure 3:**
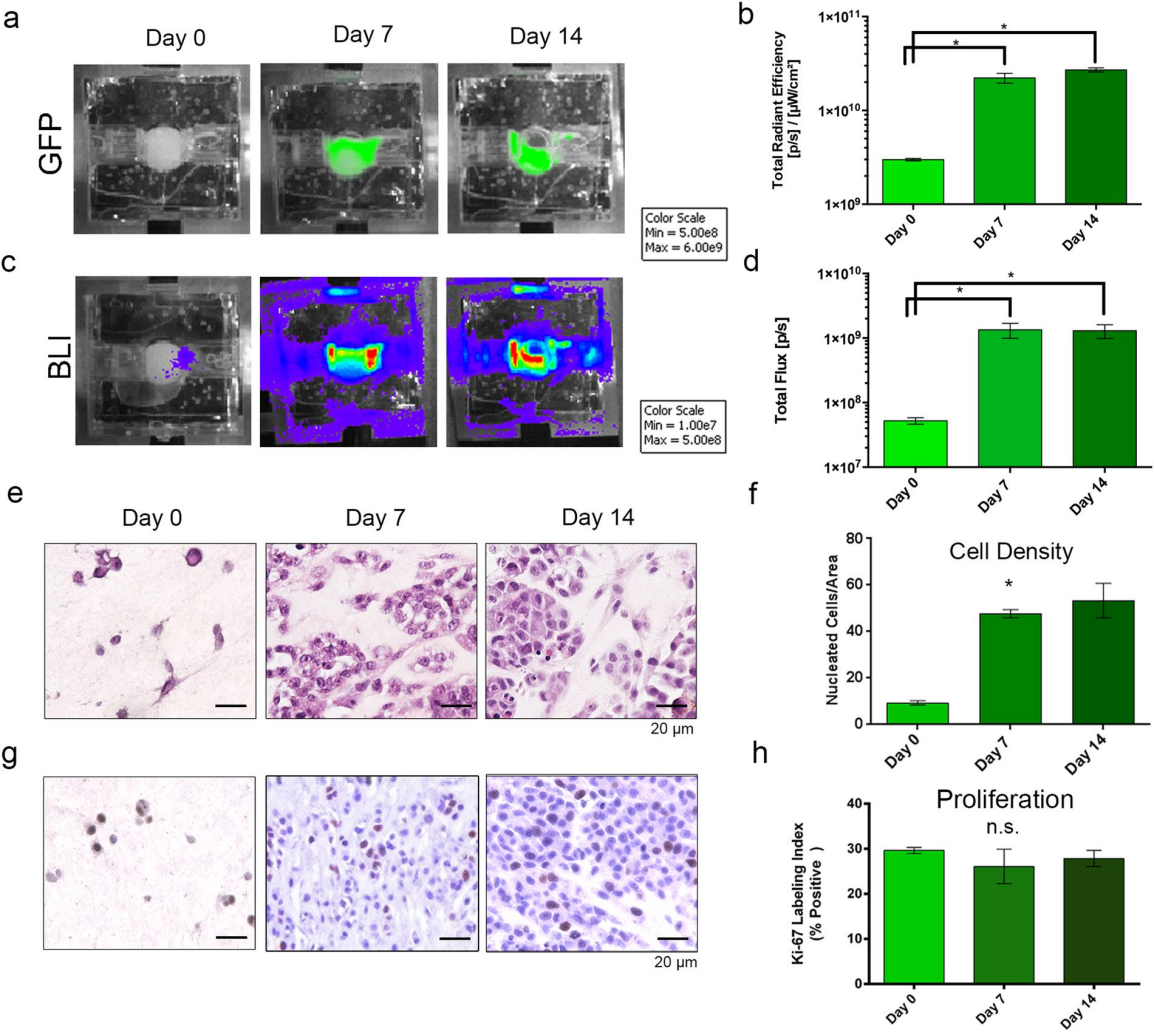
Optical Imaging to Measure Cell Growth over Time. (**a**,**c**) Representative images of fluorescence (GFP) (**a**) and bioluminescence (**c**) imaging (BLI) over 14 days of culture (TNBC model). (**b**,**d**) ROI measurements from GFP and BLI images, respectively, showing increases in signals from day 0 to day 7 or 14 (Kruskal-Wallis test, p = 0.0002 (GFP) & p = 0.0003 (BLI)). (**e**) Photomicrographs of H&E-stained histologic sections from surrogates following 0 (left), 7 (middle), or 14 (right) days growth showing increased cell density after day 0 (200x magnification). (**g**) Photomicrographs of Ki-67 immunostaining (brown nuclei) following 0 (left), 7 (middle), and 14 (right) days growth indicating stable proliferation (200x magnification). (**f**) Measurement of cell density (number of nucleated cells per cross-sectional area) from H&E-stained histologic cross-sections of surrogates imaged for global GFP and BLI levels showing a similar trend as the optical imaging methods, with the majority of cell growth occurring over the first 7 days of culture (Kruskal-Wallis test, p = 0.039). (**h**) Ki-67 labeling index from surrogates imaged for global GFP and BLI levels show stable proliferation over the culture period (Kruskal-Wallis test, not significant). For optical imaging, n = 3–9 replicate surrogates per time point. Histologic analyses were completed on 3 replicate surrogates per time point. Data in b, d, f, & h represent mean ± SE.

Next, to demonstrate the ability of the IVIS to measure cell growth over time in the surrogates, changes in global GFP and BLI were evaluated by repeated imaging of perfused TNBC surrogates over 14 days. GFP (Fig. 3a,b) and BLI (Fig. 3c,d) were measured (as described above) prior to perfusion (day 0) and on days 7 and 14, with perfusion temporarily suspended to allow for imaging. When ROI were measured, significant increases in both GFP (Fig. 3b, p = 0.0002, Kruskal-Wallis test, n = 3–7 per time point) and BLI (Fig. 3d, p = 0.0003, Kruskal-Wallis test, n = 3–7 per time point) were found between days 0 and 7, followed by a leveling off of signal between days 7 and 14, indicating the majority of cell growth occurs over the first 7 days.

To confirm the results found with IVIS imaging, 3 TNBC surrogates at each time point were processed for histologic endpoint analysis to measure cell density (the number of nucleated cells per cross-sectional area, as determined from H&E-stained histologic sections). Similar to the GFP and BLI signals, the largest increase in cell density was detected in the first 7 days of culture (Fig. 3e,f, p = 0.039, Kruskal-Wallis test, n = 3 per time point). The increase in histologic cell density correlated strongly with both GFP (r^2^ = 0.99, p = 0.029, Pearson Correlation Coefficient) and BLI signals (r^2^ = 0.97 p = 0.027, Pearson Correlation Coefficient). Analysis of cell proliferation was completed by immunohistochemical staining for Ki-67 in the same TNBC surrogates subjected to cell density analysis (Fig. 3g). No significant change in the mean Ki-67 labeling index (percentage of cells with positive staining) at each time point was found (Fig. 3h), indicating sustained cell proliferation throughout culture.

**Figure 4:**
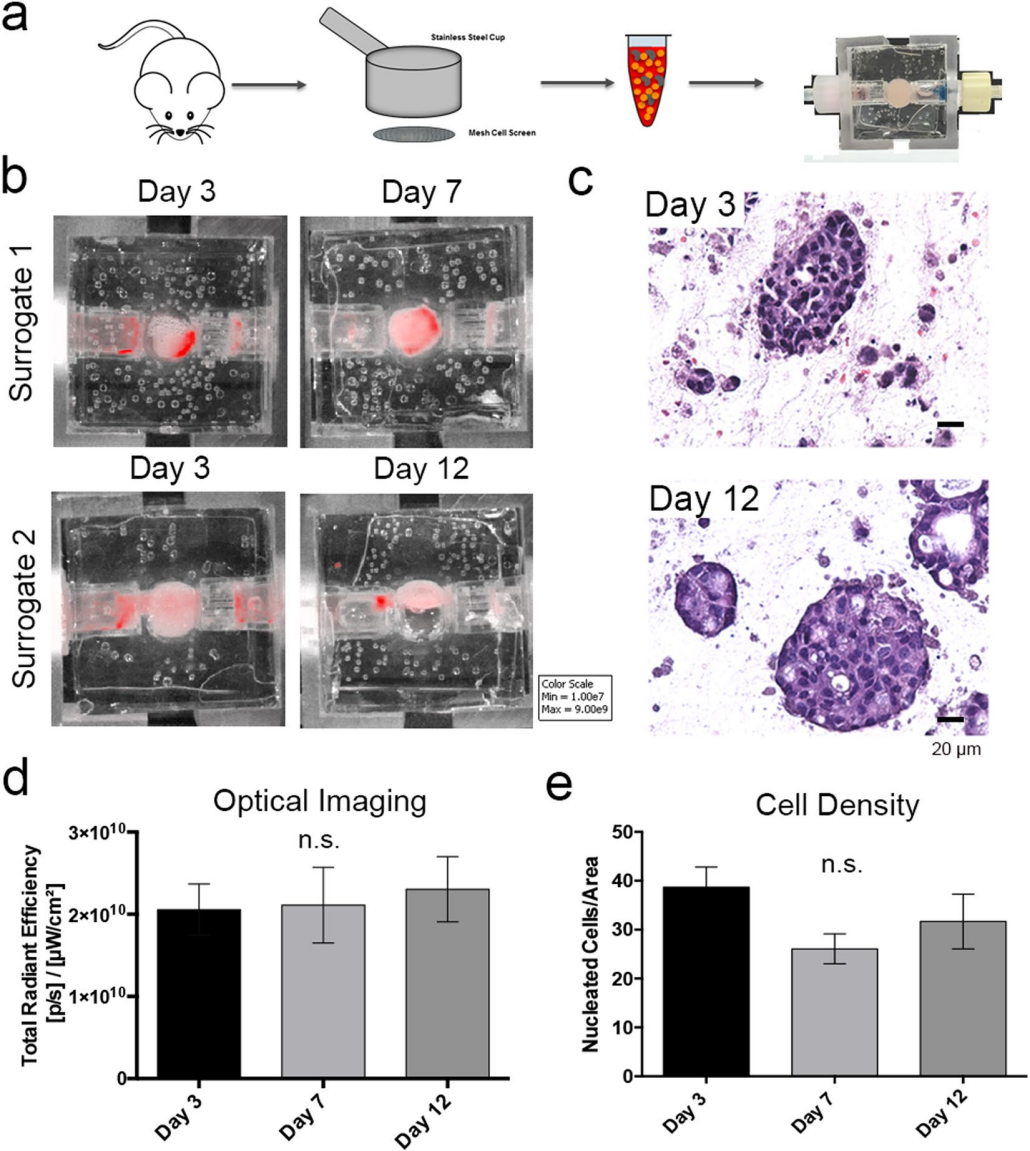
Optical Imaging of Growth over Time in Primary Culture Models (IR-783). (**a**) Schematic of surrogate setup utilizing primary murine mammary tumors (MMTV-neu), including dissociation of 0.5 g of tumor with a sieve, incorporation of dissociated cells into ECM, and placement into the perfusion bioreactor system. (**b**) Representative images from IVIS imaging of IR-783 signal in two surrogates - at days 3 and 7, or days 3 and 12 of culture - showing a stable signal over the culture period. Some variability in signal between the two surrogates at day 3 can be explained by a difference in cellularity and cell distribution in the volume of tumor incorporated into each. (**c**) Photomicrographs of H&E stained sections (200x magnification) from imaged surrogates containing murine primary tumors demonstrating an epithelial morphology. (**d**) ROI measurements of near-IR signal on days 3, 7, and 12 indicating no significant change over time (Kruskal-Wallis test, p = 0.71). (**e**) Measurement of histologic cell density evaluated as the number of nucleated cells per cross-sectional area showing maintenance of cell number over time (Kruskal-Wallis test, p = 0.22). n = 6 surrogates per time point (obtained from 3 murine tumors, 2 surrogates per murine tumor. Data in d & e represent mean ± SE.

IVIS imaging was also used to monitor the *ex vivo* maintenance of murine MMTV-neu mammary carcinomas in our surrogate/bioreactor system. *Ex vivo* culture of cancers (either murine or human) is of value in accurate modeling of disease^43–45^. However, the constituent cells of these cancers may not be amenable to induced expression of fluorescent or bioluminescent markers. To circumvent this possibility, a non-cytotoxic, near infrared heptamethine cyanine dye, IR-783, was used. IR-783 was chosen due to reports of selective uptake and retention in cancer cells without the need for chemical modification^46,47^. The excitation and emission spectra of this dye can be imaged using IVIS (Ex: 780/Em: 845), making it a candidate for testing in this application. MMTV-neu carcinomas were resected, approximately 0.5 g of each tumor was dissociated using a cell dissociation sieve, and incorporated into tumor surrogates (Fig. 4a). Following ECM polymerization, dye was injected into the surrogate volume via the upstream bioreactor port and incubated statically for 30 minutes. An initial study to determine the washout period required prior to imaging for an appropriate signal to background ratio was performed and indicated that a washout period of 3 days post incubation was optimal. Following washout, surrogates were imaged on day 3. Imaging was repeated, including re-incubation with dye and washout, on day 7 or day 12 (Fig. 4b) and was followed by fixation and processing of surrogates for histologic analysis (Fig. 4c). ROI analysis of optical images indicated maintenance of cellularity within tumor surrogates over time (Fig. 4d). Subsequent histologic assessment of cell density, as described above, confirmed that the bioreactor/surrogate system supported the *ex vivo* maintenance of the tumor cells (Fig. 4e).

### Non-invasive confocal imaging of cancer surrogates was used to evaluate changes in cell morphology and population dynamics over time

**Figure 5:**
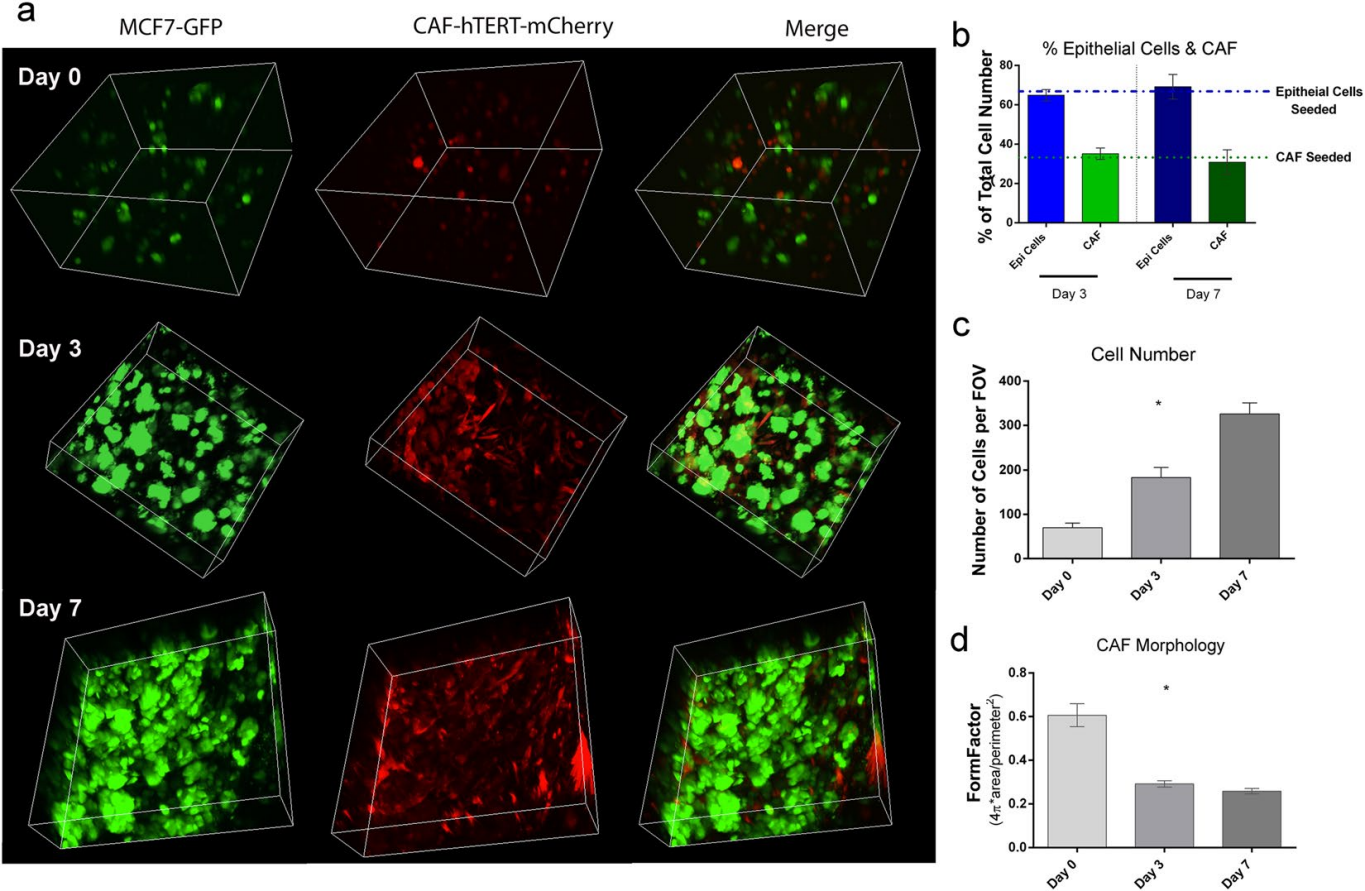
Confocal Microscopy to Evaluate Cellular Morphology and Population Dynamics. (**a**) Multiphoton confocal images (3D renderings) of ER+ tumor surrogates at days 0, 3, & 7, showing the GFP positive MCF-7 cells alone (green, left panels), the mCherry positive CAF-hTERT (red, middle panels), and both cell types together (merge, right panels) (250x magnification, 3D reconstructions are between 150 and 400 μm in thickness). (**b**) Epithelial to fibroblast ratio (green: red) was calculated from confocal maximum projections using CellProfiler and showed no significant change over time (Kruskal-Wallis test). The percentage of each cell population seeded (initial E:F = 2:1) is indicated by the dashed lines. (**c**) Total cell number (epithelial cells and CAF) was calculated from confocal maximum projections using CellProfiler and demonstrated an increase over time (Kruskal-Wallis test, p < 0.001, n = 4–7 FOV). (**d**) FormFactor, a measure of cellular circularity was calculated from confocal images using CellProfiler, with changes in CAF over time indicating cellular elongation (Kruskal-Wallis test, p < 0.0001, n = 4–7 FOV). Data in (**b**–**d**) represent mean ± SEM.

Cellular morphology and real-time evaluation of specific cell populations in the bioreactor/cancer surrogate system can be assessed at a microscopic level via multi-photon confocal microscopy. The benefits of this form of microscopy are twofold - allowing for good resolution in the z-dimension, a crucial feature for thick TE models, and little phototoxicity due to the high wavelength of the laser which can excite multiple fluorophores simultaneously^48,49^. The bioreactor was designed to accommodate confocal microscopy using long working distance objectives by including glass coverslips on upper and lower surfaces. Repeated confocal microscopy was used to evaluate changes in cell morphology, *i*.*e*., cell elongation, as well as quantitative changes in total cellularity and cell populations, *i*.*e*., ratio of cancer epithelial cells (green) to CAF (red) (E:F), over time, with the same ER+ surrogate imaged on days 0, 3, and 7 of culture (Fig. 5a). Monitoring the E:F during culture helps to ensure a recapitulative ratio is maintained. Using multi-photon confocal microscopy we are able to show that there is no significant change in the E:F over 7 days of culture in the ER + surrogate (Kruskal-Wallis test, n = 4–7 FOV per time point, Fig. 5b). The change in total cell number over time was evaluated within the fields of view (FOV) chosen using CellProfiler. A steady increase in cell number was found over the 7 days of culture (Fig. 5c) (Kruskal-Wallis, p < 0.0001, n = 4–7 FOV per time point). Visual changes in cellular morphology, specifically cell elongation, was also quantified. An elongate morphology is characteristic of fibroblasts that have adhered to a substrate. Elongation was measured by evaluating the average FormFactor (CellProfiler), a measure of cellular circularity ((4π*area)/perimeter^2^), of each cell population. With this measurement a perfectly circular cell would have a FormFactor equal to 1; therefore, more elongate cells have a lower FormFactor. The CAF showed a significant decrease in the average FormFactor, indicating cell elongation, between days 0 and 3, and days 0 and 7, (Fig. 5d, Kruskal-Wallis test, p < 0.0001, n = 4–7 FOV per time point). The changes over time were quantified using CellProfiler (3 non-overlapping areas imaged and quantified per surrogate per time point). The presence of an elongate cell population supports the adhesion and persistence of the CAF in the surrogates.

### Non-invasive IVIS imaging quantified surrogate response to therapeutic intervention

**Figure 6:**
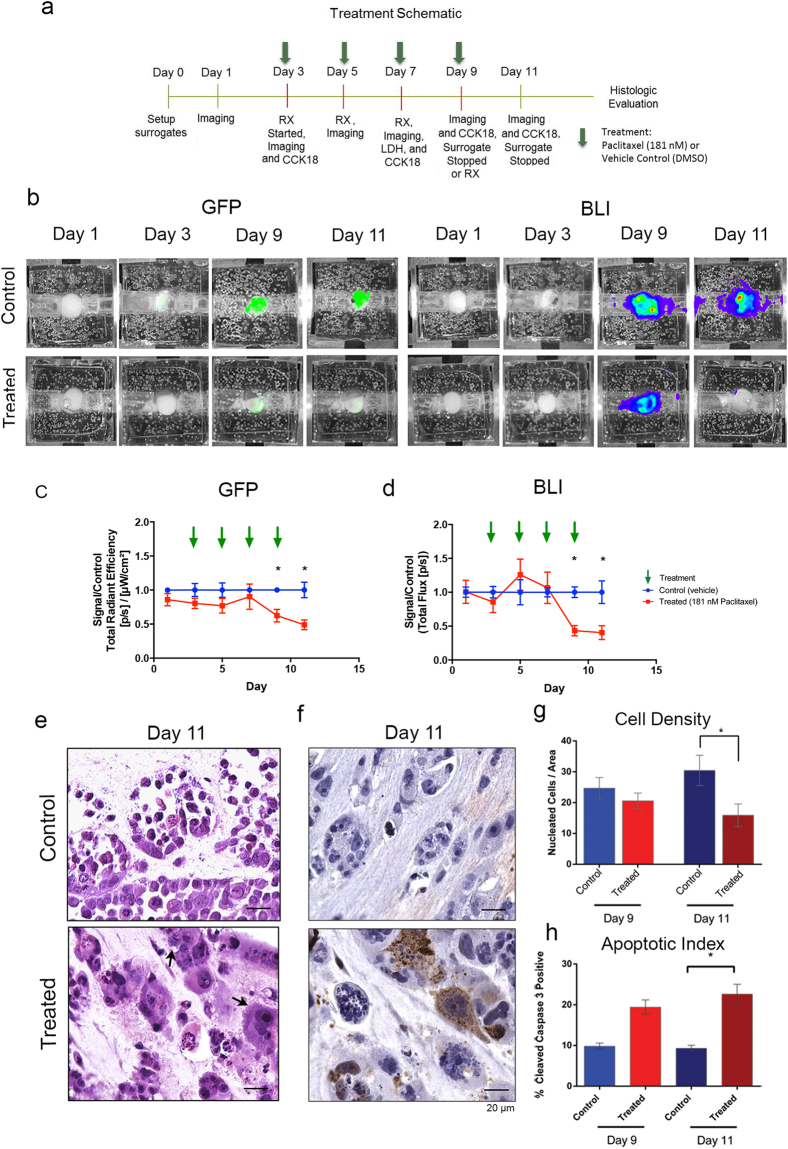
Measurement of Cell Death in Response to Therapeutic Intervention: (**a**) Treatment schematic utilized in TNBC surrogates. (**b**) Representative images of IVIS imaging (GFP (left) and BLI (right)), on days 1, 3, 9, and 11 of culture with lower signals in treated surrogates compared to control surrogates on days 9 and 11. Images obtained using the same color scale (minimum and maximum epi-fluoresence or bioluminescence measurements) at each time point. (**c**) ROI measurement of GFP in treated and control surrogates (signal in treated surrogates is normalized to the average signal of control surrogates at each time point) over time confirming lower signals at days 9 and 11 in treated surrogates (Kruskal-Wallis test, p = 0.0009 at days 9 and 11. n = 5–10 replicate surrogates per time point per condition). (**d**) ROI measurement of BLI in treated and control surrogates (signal in treated surrogates is normalized to the average signal of control surrogates at each time point) confirming lower signals at days 9 and 11 in treated surrogates (Kruskal-Wallis test, p = 0.0188 at days 9 and 11. n = 5–10 replicate surrogates per time point per condition). (**e**) Photomicrographs of H&E stained histologic sections from day 11, control (top) and treated (bottom) demonstrating cell enlargement, atypical mitotic figures and multinucleation (arrows) in treated surrogates consistent with the effect of paclitaxel (200x magnification). (**f**) Photomicrographs of cleaved caspase 3 immunohistochemical staining (brown nuclei) at day 11, control (top) and treated (bottom), showing more apoptosis in treated surrogates (200x magnification). (**g**) Measurement of cell density from histologic cross-sections shows decreased cellularity in treated versus control surrogates at day 11 (Kruskal-Wallis test, p = 0.0159, n = 4 per condition). (**h**) Measurement of apoptotic index, determined from cleaved caspase 3 staining, is greater in treated than control surrogates at day 11 (Kruskal-Wallis test, p = 0.0006, n = 4 per condition). Data in (**c**,**d**,**g**,**h**) represent mean ± SE.

Triple negative breast cancer surrogates, prepared as described above, were treated with paclitaxel, a chemotherapeutic agent frequently used in the treatment of breast cancer, or the vehicle control (DMSO) following the treatment schedule detailed in Fig. 6a. IVIS imaging (GFP and BLI) occurred on days 1 and 3, prior to treatment, and then every other day with treatment until surrogate fixation on day 9 or 11 (Fig. 6b). ROI measurements of both GFP (Fig. 6c, n = 5–10 per time point) and BLI (Fig. 6d, n = 5–7 per time point)), show a decrease in signal, indicating fewer cells present, two days after the third treatment (day 9) in paclitaxel treated surrogates compared to controls (GFP: p = 0.0009, BLI: p = 0.0188, Kruskal-Wallis test). This response was heightened two days after a fourth treatment (day 11, GFP: p = 0.0009, BLI: p = 0.0188, Kruskal-Wallis test). When H&E-stained histologic cross-sections were evaluated as an endpoint analysis (day 9 or 11), treated surrogates were found to contain cells with abnormal mitotic figures and mutinucleation (Fig. 6e) consistent with inhibition of mitotic spindle dynamics by paclitaxel. Histologic cell density was lower in treated surrogates when compared to controls at day 11 (Fig. 6g, Mann Whitney test, p = 0.0159, n = 4). Immunohistochemical staining for cleaved caspase 3 (CC3) was also completed to evaluate the apoptotic index (percentage of CC3-positive cells) in paclitaxel treated and control surrogates (Fig. 6f). The apoptotic index was higher in treated surrogates compared to controls at day 11 (Fig. 6h, Kruskal-Wallis test, p = 0.006, n = 4). Immunohistochemistry for GFP (to identify cancer epithelial cells) and fibroblast activation protein (FAP) was also performed to confirm the persistence of both cell types at day 11 (Supplementary Fig. S2).

### Measurement of circulating lactate dehydrogenase (LDH) and caspase cleaved keratin 18 (CCK18) indicated therapeutic response

**Figure 7:**
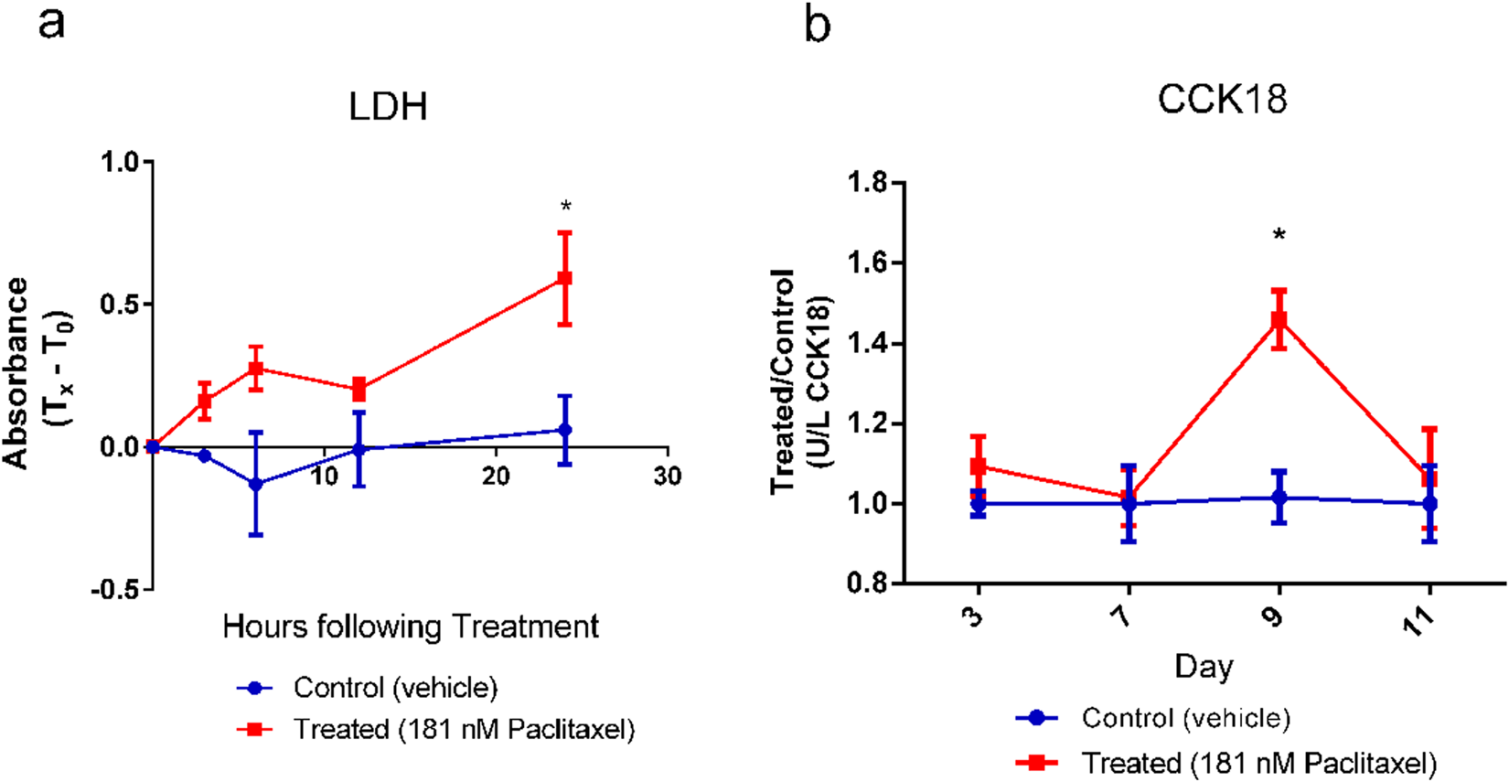
Perfusate Measurements of Cell Death in Response to Paclitaxel Treatment. (**a**) LDH assay results (total cytotoxicity) following treated and control TNBC surrogates over 24 hours (following the third treatment) showing an increase in cell death in treated surrogates compared to controls 24 hours post treatment. Data are the value at time 0 (T_0_) subtracted from all other time points (T_x_) (Kruskal-Wallis test, p = 0.0009, n = 4 per condition). (**b**) CCK18 ELISA results show increased epithelial apoptotic cell death at day 9 (following the third treatment) in treated compared to control TNBC surrogates (data for treated surrogates is normalized to controls) (Kruskal-Wallis test, p < 0.0001, n = 5–9 per condition, per time point). Data represent mean ± SE.

LDH assays and a CCK18 ELISA were performed on the perfusates from treated and control TNBC surrogates described above, to determine total cell death and epithelial cell specific death, respectively. Upon breakdown of the plasma membrane, LDH is released from cells into circulation; therefore, this measure is indicative of total cell death of both cell populations included in the surrogates^50^. LDH assays were performed on the perfusates collected at 0, 2, 4, 12, and 24 hours following the third treatment. LDH levels were higher in perfusates from treated surrogates compared to controls at 24 hours post treatment indicating an increase in cell death (total cytotoxicity) following treatment (Fig. 7a, Kruskal-Wallis test, p = 0.0009, n = 4). During apoptosis keratin 18 present in epithelial cells is cleaved by caspases^51^. Following cleavage, fragmented keratin 18 is release into circulation. Previous reports indicate that CCK18 can be used to determine epithelial cell specific apoptosis in circulating blood and media^52–57^. Results comparing CCK18 levels in perfusates from treated and control surrogates over time indicated an increased level of epithelial apoptotic death in the treated surrogates following the third treatment (day 9) (Fig. 7b, Kruskal-Wallis test, p = 0.0172, n = 5–9). This was corroborated by the response seen at this time point with IVIS imaging (Fig. 6b–d) and CC8 immunostaining (Fig. 6f,h). However, there was no difference in CCK18 between treated and control surrogates after the fourth treatment (day 11). This is likely a result of the decrease in total cells remaining after the last treatment (as indicated by the significantly lower GFP and BLI signals and cell density (Fig. 6) in treated surrogates compared to controls), since the level of circulating CCK18 is dependent on total cell number. When the day 11 CCK18 level was normalized to the day 11 GFP signal or cell density, thereby accounting for variations in cell number, a significant increase in CCK18 was found in treated surrogates compared to controls (Supplementary Fig. S3, Mann Whitney test, p = 0.0159 when normalized using GFP signal or cell density).

## Discussion

A variety of 3D *in vitro* models have been developed to evaluate specific biological processes driving breast cancer development, metastasis, dormancy, and immune modulation. These are typically non-perfused, solid 3D cultures, consisting of cancer cells embedded in ECM or cellular spheroids suspended in medium^43,58,59^. More recently, small volume 3D cultures of cancer cells, variably embedded in ECM and with or without stromal cells, have been cultured in microfluidic perfusion platforms^60–67^. Additionally, the culture of thin slices of cancer tissues, used as *in vitro* 3D models, has been explored^60–65,68^. For commercial drug development, 2D culture is still the standard culture method used in high throughput screening of compounds^69^. 3D culture is infrequently utilized in drug development, but cellular spheroids are the preferred 3D culture method in this setting^43,59,70^. Our TE surrogate system differs from other 3D systems by the larger dimension of the surrogates.

The increased dimensionality achieved with our model and other 3D models can impede standard methods of cellular analysis, such as quantification of chromogenic, fluorescent, or luminescent indicators of cell viability or death. For example, protocols based on commonly used reagents to measure cellular metabolic activity, *e*.*g*., resazurin or tetrazolium salts, are designed for use in 2D cultures. Accurate quantification of the resulting chromogenic or fluorescent signals from viable cells embedded in a 3D ECM, provided the reagents can reach the embedded cells to begin with, is problematic and requires protocol modification and standardization. Furthermore, some of these reagents are toxic to cells and do not allow real-time and repeated evaluation during culture, restricting analysis to the terminal experimental time point. We have demonstrated the utility of several non-destructive methods of evaluation of growth and viability, including optical (IVIS) imaging, confocal imaging, and analyses of perfusate/circulating culture medium that can be utilized to monitor 3D cultures or tissue surrogates over time.

A distinct advantage of optical imaging is the ability to quickly capture a global readout of cell growth or viability in the 3D culture. In contrast, methods that rely on microscopy can only capture focal regions within engineered tissue surrogates. Furthermore, this method can be used repeatedly to monitor cell growth during culture. Other methods to evaluate all or most cells in tissue surrogates, such as flow cytometry, are destructive and can therefore only be used as a method of endpoint analysis. The signals resulting from GFP and BLI imaging are not identical because different molecules and molecular processes within cells are being measured. BLI is likely a better indicator of cell viability, as the luminescent signal is dependent on the presence of ATP in metabolically active cells. For the BLI signal, the luciferase protein catalyzes the oxidation of reduced luciferin in the presence of adenosine triphosphate (ATP) and oxygen, generating carbon dioxide (CO_2_), adenosine monophosphate (AMP), pyrophosphate (PPi), oxyluciferin, and light^71,72^. However, GFP expressed by cells has a half-life of approximately 26 hours and may persist after recent cell death^73^. The major disadvantage associated with optical imaging is the requirement of labeled cells. This can be overcome by utilizing dyes that are retained by the cell types of interest, such as IR-783 used here^47^. Another disadvantage associated with optical imaging is the inability to evaluate individual cells and evaluate morphologic changes. To overcome this, multi-photon confocal microscopy can be utilized.

The depth of our engineered surrogate prevented the use of traditional fluorescence microscopy due to limited resolution in the z-dimension. Multi-photon confocal microscopy, with the use of long working distance objectives, allowed imaging into the depth of the surrogate volume without losing resolution. Using this imaging modality, each cell population could be evaluated and monitored in real-time over the course of an experiment. This imaging modality could also be used to monitor cell death, drug uptake, hypoxia, and a variety of other cellular responses^74–77^. However, only a portion of most larger, engineered, tissue surrogates can be evaluated feasibly using high resolution microscopic imaging, which could create a sampling bias, particularly when examining rare events.

Optical imaging was also useful in measuring therapeutic response to treatment of TNBC surrogates with paclitaxel. Additionally, we demonstrated the feasibility of monitoring therapeutic response by measuring LDH and CCK18 in the circulating media or perfusate. Commercial kits have been developed to measure LDH in 2D *in vitro* culture, yet measurement of LDH in 3D or TE *in vitro* systems is not common. LDH is a measure of total cell death and, in complex surrogates with multiple cells types, will indicate death of all cell types affected by the treatment. To measure death of the cancer epithelial cells only, we assayed for CCK18, an epithelial cell specific measurement of apoptotic cell death^51^. One caveat of the LDH and CCK18 assays is that they are dependent on cell number and the time required for the cells to respond to treatment. Therefore, if the treatment is cytotoxic and reduces the cell number below the control, the released LDH or CCK18 will also be lower. This was observed with the CCK18 level, which was lower in the perfusates of treated surrogates at day 11 than day 9 and similar to control surrogates at day 11, at which point (day 11) the cell number present (measured by cell density and GFP) is significantly lower in the treated than control surrogates. When the variation in cell number is taken into account, the treated surrogates had a significantly higher CCK18 level, compared to controls. These measurements indicate that the majority of cell death in response to treatment is occurring following the third treatment (between days 7 and 9). Therefore, these perfusate measurements may be considered earlier indicators of response, compared to histologic evaluation. Another caveat of these perfusate assays is that the treatment must be cytotoxic, as cytostatic therapies do not facilitate release of LDH and CCK18^78^. Furthermore, these assays require a time course study with each model system and candidate therapeutic in order to determine the sensitivity and optimal time points for measurement after treatment.

## Conclusion

Despite our growing understanding of the importance of the tissue microenvironment in determining cancer behavior and response to treatment, 2D culture models remain the “gold standard” for preclinical drug discovery and development. A variety of factors contribute to the lack of adoption of complex 3D models, one of which is the challenge of accurately evaluating cellular and molecular responses in these models. Identification and standardization of more efficient and accurate analytical techniques to evaluate and validate these systems will hasten the adoption of 3D models as platforms for biomedical research and pharmaceutical development. The work presented identifies several methods of assessing cell growth and response to treatment and provides an approach to optimizing these methods for use in TE 3D models.

### Methods

#### Cell culture

MDA-MB-231 cells were obtained from Dr. Danny Welch (University of Kansas) and subsequently transduced with GFP/LUC (CMV-luciferase-ires-puro.T2A.GFP) (231-GFP/LUC). MCF-7 cells were obtained from American Type Culture Collection and subsequently transduced with GFP/LUC (MCF7-GFP/LUC). Breast cancer associated fibroblasts (CAF) were isolated from remnant breast cancer tissues by us as described^79^, after approval from the University of Alabama at Birmingham (UAB) Institutional Review Board for Human Use (IRB) and in accordance with all IRB and institutional guidelines and regulations. Subsequently, CAF were immortalized via transduction of human telomerase (CAF-hTERT) as described^41^. CAF-hTERT were also transduced to express mCherry using retroviral particles (Geneacopeia). 231-GFP/LUC were maintained in Dulbecco’s Modified Eagle’s Medium (DMEM, Corning) supplemented with 10% Fetal Bovine Serum (FBS, Atlanta Biologicals) under the selection of 20 µg/ml puromycin (MP Biologics). MCF7-GFP/LUC were maintained in Modified Eagle’s Medium (MEM, Corning) supplemented with 10% FBS, 0.01 mg/ml insulin (Sigma Aldrich), under the selection of 10 µg/ml puromycin. CAF-hTERT were maintained in DMEM supplemented with 10% FBS and 10 µg/ml hygromycin (MP Biologics). CAF-hTERT-mcherry were cultured similarly, under the selection of hygromycin (10 µg/ml) and puromycin (2.5 µg/ml).

#### Perfused surrogate preparation

231-GFP/LUC or MCF7-GFP/LUC and CAF-hTERT(+/− mCherry) (2:1 epithelial to fibroblast (E:F) ratio, 5.25 × 10^5^ total cells/100 µl ECM) were mixed into an ECM containing 50% bovine fibrin (Sigma Aldrich) +45% bovine collagen I (Advanced Biomatrix) +5% basement membrane (growth factor reduced Matrigel (BM), Corning) - total protein approximately 6 mg/mL- and injected into a polydimethylsiloxane (PDMS) bioreactor (Fig. 1B). This volume was perforated by five 400 μm Teflon coated wires located within an upstream wire-guide. Alternatively, primary mouse mammary tumor tissue (approximately 0.5 g), resected from the MMTV-neu model of mammary carcinoma, were dissociated through a tissue dissociation sieve (Sigma Aldrich, 280 μm pore size) and the cellular component was incorporated into the ECM before injection into the bioreactor, as described above. The cell suspension from each murine tumor was split between 7 bioreactors (cultured for 1, 3, 7, or 12 days). Following ECM polymerization, Teflon wires were removed, generating five microchannels in the ECM/cell mixture. Surrogates were connected to a micro-peristaltic pump and a media reservoir via peroxide cured silicone tubing (Cole Parmer), as previously described^41^, and continuously perfused with 15 mL medium (HMMEC media containing 5% FBS and antibiotics for cell line surrogates or mammary epithelial media for murine tumor surrogates (Phenol Red Free DMEM/F12 supplemented with 10% FBS, 20 ng/ml EGF, 0.5 μg/ml hydrocortisone, 100 ng/ml cholera toxin, 100 μg/ml bovine insulin, and antibiotics) for 1 to 14 days (37 degrees, 5% CO2), with medium changed every 3 days.

#### IVIS imaging

IVIS-100 and IVIS Lumina imaging systems were used to non-destructively image global fluorescence and bioluminescence (BLI) of surrogates. Surrogates were sterilely disconnected from the flow loop and closed to surroundings in a laminar flow hood. Fluorescent signal of GFP positive cells was imaged using GFP excitation and emission filter sets (Ex: 460/Em: 520), with 1 second exposure, bin 2, f/stop 2. Luminescence signal of luciferase positive cells was imaged 8 minutes following injection of 1 mL d-luciferin (XenoLight D-Luciferin Potassium Salt, Perkin Elmer, 5 μg/mL); similar imaging settings were used for each experiment. Identical square regions of interest (ROI) were drawn around surrogates to measure GFP and BLI signals, using the largest surrogate to determine ROI size for the entire experiment (as detailed in Supplementary Fig. S4). The same ROI was used to evaluate the GFP or BLI signals within an experiment.

#### IR-783 incubation and imaging

Following matrix polymerization of murine tumor surrogates, 20 μM IR-783 was injected into the microchannels and incubated statically for 30 minutes (37 degrees, 5% CO2), as in prior reports^46^. After dye incubation, surrogates were perfused with Hank’s balanced salt solution (HBSS) to wash, and then mammary epithelial cell media was added and perfused for a 3 day washout period prior to imaging. Surrogates were imaged with the IVIS Lumina on day 3 (Ex: 780/Em: 845), and on day 7 before fixation or day 12 before fixation with a re-incubation of dye 3 days prior to imaging. Square regions of interest (ROI) were drawn around surrogates to measure IR-783 signal.

#### Histologic processing and immunohistochemistry

Following growth, surrogates were fixed with neutral buffered formalin, processed to paraffin, and histological sections were prepared, as previously described^3^. Sections were stained with hematoxylin and eosin (H&E) to evaluate cellular morphology and cell density (number of cells per cross-sectional area), and immunohistochemistry was performed to detect Ki-67 (1:100, clone Sp6, Thermo Scientific), cleaved caspase 3 (1:100, clone D3E9, Cell Signaling), GFP (1:100, clone D5.1, Cell Signaling), or FAP (1:150, clone EPR20021, Abcam) using the Dako Envision + Dual Link secondary detection kit containing the chromogen DAB, following antigen retrieval (10 mM citrate buffer, pH 6, Biogenex or Tris-EDTA, pH 9 (for anti-FAP staining only)).

#### Image analysis of histologic sections for cell density

Cell density, defined as the number of nucleated cells per 1 × 10^6^ pixels^2^ (area), was determined from photomicrographs (400x) of H&E-stained histologic sections of surrogates (complete cross sections of each surrogate analyzed). The number of nucleated cells was either counted manually or determined using CellProfiler analysis^80–82^, as previously published^40^. The number of nucleated cells was normalized to the cross-sectional area of each surrogate, measured using the polygon tool in ImageJ.

#### Confocal Microscopy & Analysis

Multi-photon confocal microscopy was completed on surrogates containing MCF-7-GFP/LUC and CAF-hTERT-mCherry using a Nikon A1R multi-photon confocal laser scanning microscope with a 25x objective (Nikon Apo LWD 25x/1.10 W). A pulse laser at 830 nM was used to excite both GFP and mCherry. Z-stacks from 2–4 fields of view per bioreactor were imaged at each time point. The maximum intensity projection of each z-stack (per channel) and the 3D rendering (combined channels) were obtained using Nikon Elements. CellProfiler was used to evaluate the cell number (per cell type), as previously published, and the degree of cell elongation (measuring the FormFactor, defined as 4πArea/Perimeter^2^) from the maximum intensity projections of each z-stack (per channel).

#### Paclitaxel treatment and perfusate measurements

*Paclitaxel treatment* *-* Paclitaxel (Acros Organics) or vehicle control (DMSO) were diluted in 15 mL surrogate media (HMMEC media containing 5% FBS) to a 181 nM concentration (5 times the IC_50_ found in 2D culture of MDA-MB-231) and perfused for 48 hours before retreatment. *LDH measurement* *-* Following the 3rd treatment of paclitaxel or vehicle control (day 7), 500 μL of perfusate was removed from the flow loop downstream of the surrogate 0, 3, 6, 12, & 24 hours post-treatment. Pierce LDH Cytotoxicity Kit was used, as directed, to measure the free LDH level in the circulating media in response to treatment over time. *Caspase Cleaved Keratin 18* ( *CCK18* ) *measurement*- CCK18 was evaluated using the M30 Apoptosense ELISA kit (Peviva) as directed, with perfusate diluted 1 to 4 prior to CCK18 measurement.

### Statistical Analysis

Pearson’s correlation coefficient was computed to evaluate correlations between two groups. The Kruskal-Wallis test with Dunn’s multiple comparison testing (where applicable) was used to evaluate significant differences between three or more groups. The Mann Whitney test was used to evaluate significant difference between two groups.

## Electronic supplementary material

Supplemental Data
