# Supplementary material for: Methods to Evaluate Cell Growth, Viability, and Response to Treatment in a Tissue Engineered Breast Cancer Model
Source: Sci Rep. 2017 Oct 26;7:14167. doi: 10.1038/s41598-017-14326-8 (PMC5658356; doi:10.1038/s41598-017-14326-8)
Supplement: Supplementary file 1 — Supplemental Data [file 41598_2017_14326_MOESM1_ESM.pdf]

Methods to Evaluate Cell Growth, Viability, and Response to Treatment in a Tissue Engineered Breast Cancer Model

Kayla F. Goliwas, Jillian R. Richter, Hawley C. Pruitt, Lita M. Araysi, Nicholas R. Anderson, Rajeev S. Samant, Susan M. Lobo-Ruppert, Joel L. Berry, Andra R. Frost

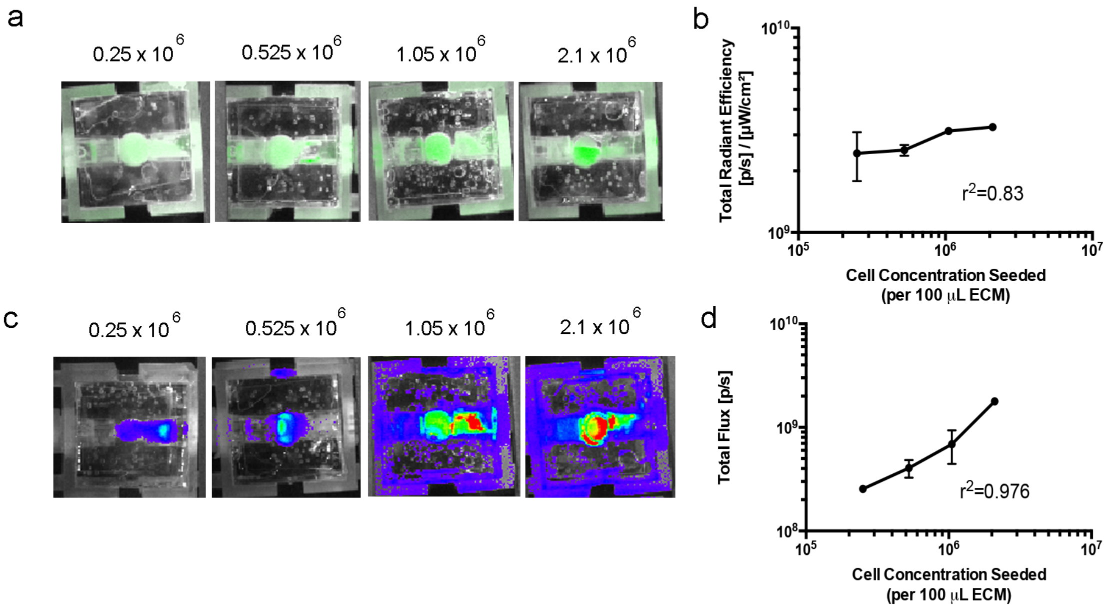

Supplemental Figure 1: Correlation of Optical Imaging with Cell Concentration within Engineered ER+ surrogates. **a** & **c**) Fluorescence (GFP) & bioluminescence imaging (BLI) of increasing cell concentrations on day 0 showing increasing signal with increasing cell concentration. **b** & **d**) Graphical representation of region of interest measurements (ROI) from GFP and BLI imaging completed on the day of surrogate setup (day 0). R^2value for correlation analysis of cell concentration seeded and signal shows a strong correlation (r^2=0.83 GFP (p=0.08), r^2= 0.98 BLI (p=0.012), Pearson correlation coefficient). n=3 replicate surrogates per cell concentration. Data in b & d represent mean ± SE.

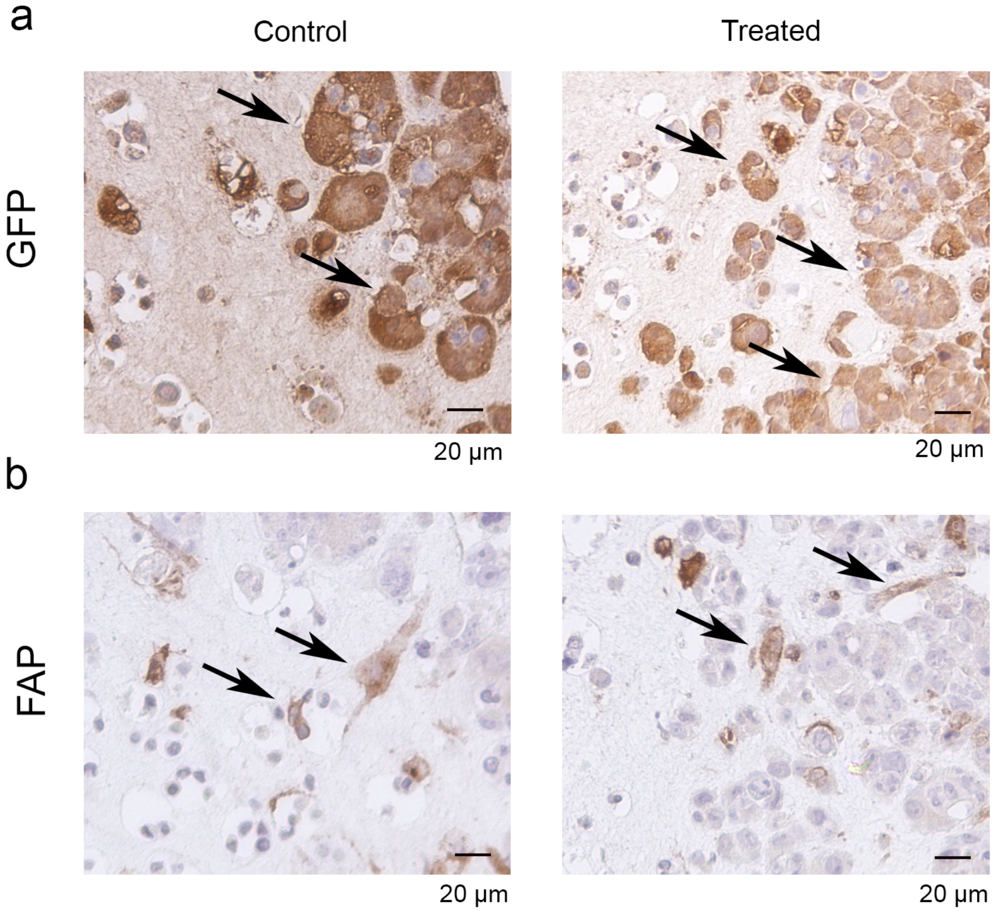

Supplemental Figure 2: Immunohistochemical Staining Showing the Presence of both Cell Populations. **a**) GFP staining indicating the presence of epithelial cells (arrows) following 11 days culture in control (left) and treated (right) surrogates (200x magnification). **b**) FAP staining indicating the presence of CAF (arrows) following 11 days culture in control (left) and treated (right) surrogates (200x magnification).

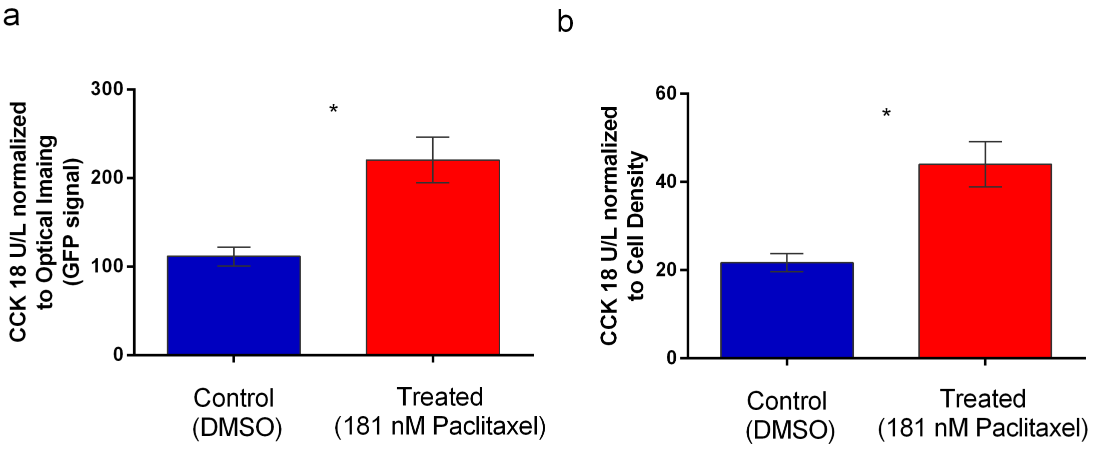

Supplemental Figure 3: CCK18 normalization to Cell Number at Time of Analysis. **a**) CCK18 levels normalized to average GFP signal at day 11 shows a significant increase in the amount of circulating CCK18 in treated surrogates compared to controls (Mann Whitney test, p=0.016). **b**) CCK18 levels within perfusates normalized to the average cell density at day 11 show a similar significant increase in treated surrogates when compared to controls (Mann Whitney test, p=0.016). n=3 replicate surrogates per condition. Data represent mean ± SE.

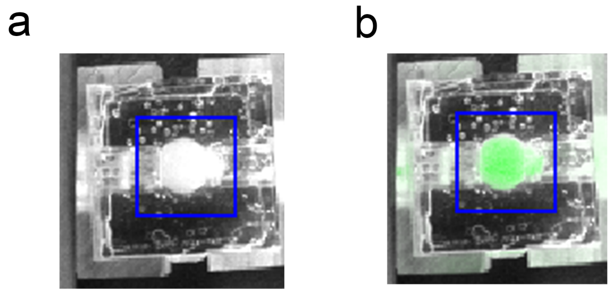

Supplemental Figure 4: Region of Interest (ROI) Selection within IVIS Images. IVIS brightfield image (**a**) and fluorescence image (**b**, Ex: 460 /Em: 520) from Fig. 1 (0.25 x 10^6 cells) showing that the ROI was selected to encompass the entirety of the largest surrogate (blue box) in the experiment to measure the global GFP signal. This same ROI was applied to all other surrogates within a single experiment.
